# Fe_3_O_4_ Nanoparticles in Combination with 5-FU Exert Antitumor Effects Superior to Those of the Active Drug in a Colon Cancer Cell Model

**DOI:** 10.3390/pharmaceutics15010245

**Published:** 2023-01-11

**Authors:** Sidika Genc, Ali Taghizadehghalehjoughi, Yesim Yeni, Abbas Jafarizad, Ahmet Hacimuftuoglu, Dragana Nikitovic, Anca Oana Docea, Yaroslav Mezhuev, Aristidis Tsatsakis

**Affiliations:** 1 Department of Medical Pharmacology, Faculty of Medicine, Bilecik Seyh Edebali University, Bilecik 11230, Turkey; 2 Department of Pharmacology, Faculty of Medicine, Ataturk University, Erzurum 25240, Turkey; 3 Faculty of Chemical Engineering, Sahand University of Technology, Tabriz 51335-1996, Iran; 4 Laboratory of Histology-Embryology, Medical School, University of Crete, 71003 Heraklion, Greece; 5 Department of Toxicology, University of Medicine and Pharmacy of Craiova, 200349 Craiova, Romania; 6 Center of Biomaterials, D. Mendeleev University of Chemical Technology of Russia, 125047 Moscow, Russia; 7 Department of Forensic Sciences and Toxicology, Faculty of Medicine, University of Crete, 71003 Heraklion, Greece

**Keywords:** Fe_3_O_4_ nanoparticles, 5-fluorouracil, Caco-2, PTEN, IL-10, oxidative status

## Abstract

(1) Background: Colon cancer is one of the most common cancer types, and treatment options, unfortunately, do not continually improve the survival rate of patients. With the unprecedented development of nanotechnologies, nanomedicine has become a significant direction in cancer research. Indeed, chemotherapeutics with nanoparticles (NPs) in cancer treatment is an outstanding new treatment principle. (2) Methods: Fe_3_O_4_ NPs were synthesized and characterized. Caco-2 colon cancer cells were treated during two different periods (24 and 72 h) with Fe_3_O_4_ NPs (6 μg/mL), various concentrations of 5-FU (4–16 μg/mL), and Fe_3_O_4_ NPs in combination with 5-FU (4–16 μg/mL) (Fe_3_O_4_ NPs + 5-FU). (3) Results: The MTT assay showed that treating the cells with Fe_3_O_4_ NPs + 5-FU at 16 µg/mL for 24 or 72 h decreased cell viability and increased their LDH release (*p* < 0.05 and *p* < 0.01, respectively). Furthermore, at the same treatment concentrations, total antioxidant capacity (TAC) was decreased (*p* < 0.05 and *p* < 0.01, respectively), and total oxidant status (TOS) increased (*p* < 0.05 and *p* < 0.01, respectively). Moreover, after treatment with Fe_3_O_4_-NPs + 5-FU, the IL-10 gene was downregulated and PTEN gene expression was upregulated (*p* < 0.05 and *p* < 0.01, respectively) compared with those of the control. (4) Conclusions: Fe_3_O_4_ NPs exert a synergistic cytotoxic effect with 5-FU on Caco-2 cells at concentrations below the active drug threshold levels.

## 1. Introduction

Colon cancer is the third most common malignancy and represents the second most common cause of cancer death despite advances in diagnosis and treatment [1]. One of the key distinguishing features of colon cancer is the loss of cellular organization and the increased ability to invade near and distant sites. The standard treatment principle encompasses chemotherapy, surgery, and radiotherapy according to the type and stage of the disease. However, the gene-type signature of the cancer tissue alters the neoplasm response to therapy regimens [2]. Thus, the 5-year survival of colon cancer patients remains at 64%, and efficient therapy, with attenuated side effects, remains a significant unmet health need [3]. 

5-Fluorouracil (5-FU), an antineoplastic agent, is used to treat colon cancer by inhibiting the S phase of the cell cycle, thereby blocking DNA synthesis and triggering cell death [4,5]. However, due to its short half-life (5–14 min), poor membrane permeability, and rapid metabolism, high doses must be continuously administered to maintain a minimum therapeutic serum concentration, which often entails numerous side effects and severe toxicity [6,7,8]. Indeed, chemotherapeutics that prevent DNA synthesis can incur lethal side effects in rapidly dividing healthy tissues, including intestinal epithelium and blood cells [9].

Numerous studies have demonstrated that NPs significantly increase the drug uptake of cancer cells, thus improving the limitations of current chemotherapeutic agents [10]. Different metal NPs, including AU, TiO, Fe_3_O_4_, Pt, ZnO, and Mg NPs, were previously tested in various cancer models [11,12]. In addition, Fe_3_O_4_ nanoparticles (Fe_3_O_4_-NPs), or magnetite NPs, exhibit magnetic properties and have lately received approval from the Food and Drug Administration (FDA) for utilization in magnetic resonance imaging (MRI) [13]. 

Notably, metal NPs can enhance the accumulation of active drugs in a passive and active manner. For example, cancer tissues exhibit leaky vasculature with pore sizes reaching hundreds of nanometers, which enables the passive accumulation of inert metal NPs [14]. This mechanism is called the increased permeability and retention (EPR) effect. Furthermore, introducing functional modifications on the metal NP’s surface will result in specific tissue targeting [15]. Indeed, incorporating specific ligands for tumor targeting, including peptides, antibodies, glycans, or folic acid, can increase drug release to tumor cells [16]. 

Notably, in some models, Fe_3_O_4_ metal oxides and their various composites [17,18] exhibit cytotoxic and genotoxic properties, resulting in DNA fragmentation, disturbance in the integrity of the mitochondrial membrane, and cell necrosis, as well as alterations in oncogene expression. 

Furthermore, Fe_3_O_4_/composite nanomaterials enhanced reactive oxygen species (ROS) production and oxidative stress-inducing cell apoptosis [19]. Another exciting aspect of iron oxide action is its immunomodulatory effects. Thus, the inflammatory response of neutrophils in a vascular mimetic model is attenuated upon the uptake of iron oxide NPs [20]. Cytokines play a critical role in regulating the host cell’s immune response to cancer and the mechanism of tumorigenesis [21]. Interleukin-10 (IL-10), an important cytokine secreted by various cell types, including macrophages, monocytes, neutrophils, and endothelial cells [22], was shown to affect the homeostasis of the intestinal epithelium. Notably, IL-10 is a crucial inhibitor of the immune response harnessed by various tumors to evade the immune system [19,23,24]. 

Phosphatase and tensin homolog (PTEN) is a protein tyrosine phosphatase expressed in humans [25]. PTEN has been characterized as a key tumor suppressor gene due to its ability to downregulate phosphatidylinositol 3-kinase (PI3K) and downstream Akt/mTOR signaling crucial to the modulation of cell growth [26,27]. Furthermore, both loss and partial/complete inactivation of PTEN expression have been identified in many cancers, allowing its characterization as an oncogene [27]. Indeed, PTEN inactivation/loss is one of the most frequent genetic alterations in sporadic cancer [27]. Heterogeneous PTEN hamartoma tumor syndrome (PHTS), due to pathogenic variants in the PTEN onco-suppressor gene, strongly correlates with colon cancer incidence [28]. Notably, in vivo colon cancer models demonstrated that efficient anticancer therapy increased PTEN expression [29]. 

In the current study, we evaluated the effects of the Fe_3_O_4_-NPs + 5-FU combination on cell viability, oxidative stress, cytokine, and oncogene expression in a Caco-2 colon cancer cell model. Our data demonstrated that the Fe_3_O_4_-NPs + 5-FU combination exerts anticancer effects at concentrations at which the active drug, 5-FU, is ineffective. Furthermore, this article shows that the combined administration of Fe_3_O_4_ nanoparticles with 5-FU without its prior immobilization significantly increased the antitumor activity and reduced the therapeutic dose of 5-FU.

## 2. Materials and Methods

### 2.1. Chemicals and Reagents 

5-FU was obtained from Deva A.S (Istanbul, Turkey). Fe (acac)3, phosphate-buffered solution (PBS), Dulbecco’s modified Eagle’s medium (DMEM), fetal bovine serum (FBS), trypsin (with EDTA), antibiotic, oleyl amine, and dibenzyl ether were supplied by Sigma-Aldrich (St. Louis, MO, USA).

### 2.2. Fe_3_O_4_ Nanoparticles Synthesis 

**Figure 1:**
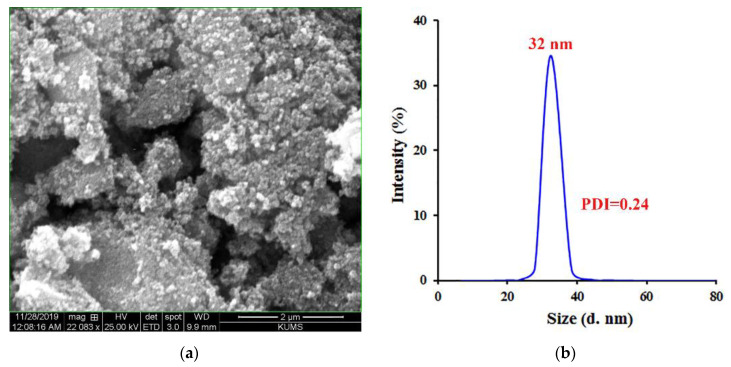
The (**a**) SEM image and (**b**) DLS analysis of Fe_3_O_4_ NPs.

Iron (III) acetylacetonate (Fe (acac)3, 1.06 g) was dissolved in a mixture of oleyl amine (15 mL) and dibenzyl ether (15 mL) under continuous stirring in a four-necked round-bottom glass reactor. The mixture was heated to 120 °C and held at the same temperature for 1 h to remove moisture under a stream of nitrogen gas. The mixing process continued throughout all stages. After one hour, the mixture temperature was rapidly increased to 300 °C, and the reaction was continued at this temperature for an 1 h. Finally, ethanol (3 × 40 mL) was added to the mixture, which was centrifuged at 8500 rpm for 12 min. After purification, Fe_3_O_4_ NPs were dispersed in hexane (10 mL). Figure 1B shows a representative SEM image of the prepared Fe_3_O_4_ NPs [30,31].

### 2.3. Fe_3_O_4_ Nanoparticles Characterization

The scanning electron microscope (SEM) images were obtained using an FEI Quanta 450 (USA). Dynamic light scattering (DLS) experiments were performed utilizing a Zetasizer Quinta Nano ZS90 (Malvern Instruments, Malvern, UK) at room temperature. Samples were prepared as 0.5% (*w*/*v*) solutions in DDW. 

The Fourier transform infrared (FTIR) spectrum of the Fe_3_O_4_ NPs was obtained with a Shimadzu 8101 M FTIR (Kyoto, Japan) using the potassium bromide (KBr) pellet technique. The powder X-ray diffraction (XRD) pattern of the Fe_3_O_4_ NPs was obtained using a Siemens D5000 diffractometer (Aubrey, TX, USA) and an X-ray generator (CuKα radiation with λ = 1.5406 Å) at room temperature [31].

### 2.4. Cell Cultures

#### 2.4.1. Caco-2 Cell Culture

Caco-2 (HTB-37™) cells were obtained from ATCC. The cells were cultured in DMEM (1% antibiotic (amphotericin B, penicillin, and streptomycin) and 10% FBS), and held at the optimum conditions (5% CO_2_; 37 °C). 

#### 2.4.2. Cell Treatments

After the cells reached 85% confluency, they were harvested and seeded in 96-well plates (Corning, Corning, NY, USA) [32]. Treatments were determined as control, Fe_3_O_4_ NPs 6 μg/mL, 5-FU (4, 8, and 16 μg/mL), and a combination (Fe_3_O_4_ NPs + 5-FU). The cells were exposed to the various treatments for 24 or 72 h.

### 2.5. MTT Assay

At the end of the experiment (after 24 and 72 h of treatment), MTT solution (10 μL) was added to each well, and the cell number was determined. In short, the plates were incubated for 4 h in a CO_2_ incubator, to which 100 µL of DMSO solution was added to all wells. The spectrophotometer read the density at 570 nm [31].

### 2.6. Total Oxidant Status (TOS) and Total Antioxidant Capacity (TAC) Determination

Total oxidant status (TOS) and total antioxidant capacity (TAC) evaluations were performed spectrophotometrically (Multiskan ™ GO Microplate Spectrophotometer reader) as previously described [26]. The color density is correlated to the oxidant levels in a sample [33].

### 2.7. Lactate Dehydrogenase (LDH) Measurement

According to the manufacturer’s instructions, the lactate dehydrogenase (LDH) was determined with an LDH detection kit. In summary, Caco-2 cells were seeded in a 96-well plate at a density of 10^3^–10^6^ cells/well in 200 μL of the medium. Six wells were prepared for each concentration. Triton X-100 (10%) and the assay buffer were added, and the wells were incubated at room temperature for one hour. After centrifugation, the cell supernatant was transferred to a new 96-well assay plate. The LDH reaction solution was added to each well, and the plate was incubated with gentle shaking on an orbital shaker for 30 min at 37 °C. A microplate reader measured the absorbance OD value at 490 nm [34]. ((experimental value A490) − (spontaneous release A490))**/**((maximum release A490) − (spontaneous release A490)) × 100. 

Maximum release: 100% dead cells by adding Triton X-100. 

Spontaneous release: nontoxic materials (cell medium) control group.

Experiment value: application groups.

### 2.8. Gene Expression Determination 

The total RNA from Caco-2 cells was used to synthesize complementary DNA (cDNA) using a high-capacity cDNA Reverse Transcription Kit. The sequences of the gene-specific PCR primers are listed below (forward and reverse). Results were compared with the control group and are expressed as relative fold. Gene expressions were normalized to beta actin using the ^ΔΔ^Ct method. 

Beat actin: CCAACCGCGAGAAGATGA′; CCAGAGGCGTACAGGGATAG′

PTEN: TGAGTTCCCTCAGCCGTTACCT′; GAGGTTTCCTCTGGTCCTGGTA′ 

IL-1β: TCTCAGATTCACAACTGTTCGTG′; AGAAAATGAGGTCGGTCTCACTA′

IL-10: GGCATGCTTGGCTCAGCACTG-3′; GCCCTGCAGTCCAGTAGACG′

### 2.9. Statistical Analyses

Statistical comparisons between the groups were calculated using one-way ANOVA and Tukey’s HSD method. All calculations were performed using SPSS 20 software for statistical analysis, and a *p* < 0.05 was considered a statistically significant difference in all tests. Results are presented as mean and standard deviation (mean ± SD).

## 3. Results

### 3.1. Characterization of Fe_3_O_4_ NPs

The synthesized Fe_3_O_4_ NPs were characterized using SEM and DLS analysis, as presented in Figure 1. The SEM image showed that the shape of the synthesized Fe_3_O_4_ NPs was spherical with an average diameter of 35 ± 5 nm. The DLS analysis revealed that the Fe_3_O_4_ NPs had an average size of ~32 nm. In addition, the polydispersity index (PDI) of the synthesized NPs was found to be 0.24, which indicated their relatively monodisperse synthesis.

**Figure 2:**
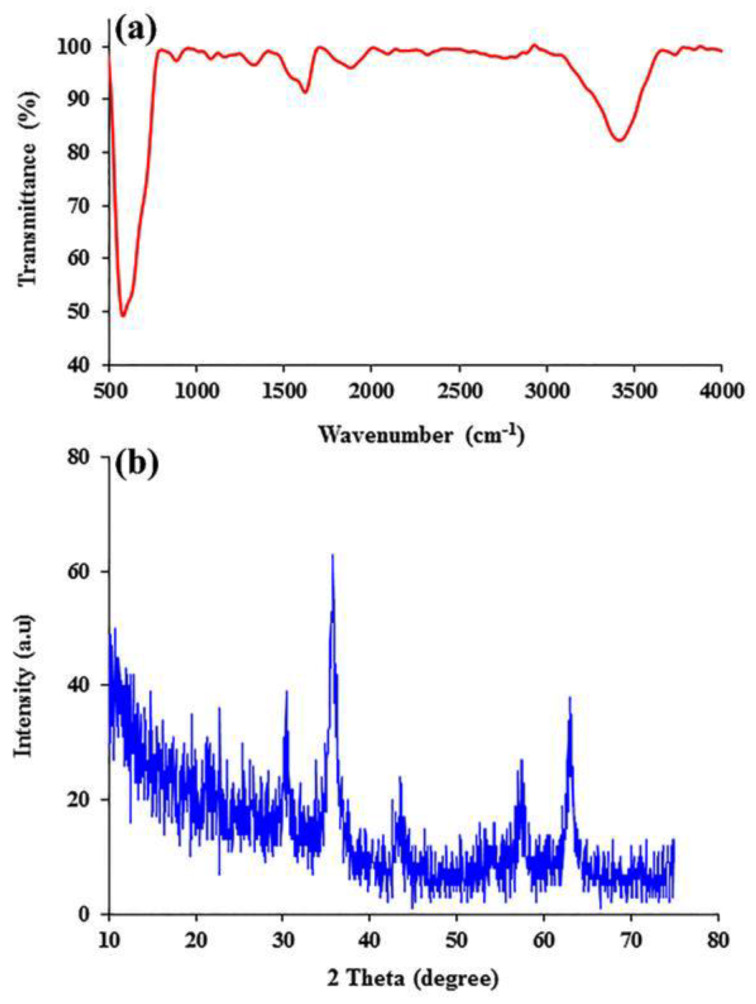
FTIR spectrum (**a**) and XRD pattern (**b**) of Fe_3_O_4_ NPs.

The FTIR spectrum and XRD pattern of the Fe_3_O_4_ NPs are shown in Figure 2. The most prominent absorption bands in the FTIR spectrum are the stretching vibration of the metal–oxygen (Fe–O) group at 576 cm^−1^ and the stretching and bending vibrations of the surface hydroxyl groups at 3420 and 1608 cm^−1^, respectively (Figure 2a).

The crystallographic analysis of the synthesized Fe_3_O_4_ NPs was performed by XRD analysis, as depicted in Figure 2b. In the XRD model of Fe_3_O_4_ NPs, the characteristic peaks belonging to the XRD spectrum at 2θ = 30.6°, 36.1°, 43.1°, 52.6°, 57.7°, and 63.1° can be indexed at (220), (311), (400), (422), (511), and (440), respectively. This FTIR spectrum and XRD pattern confirm the successful synthesis of Fe_3_O_4_-NPs.

### 3.2. Evaluation of Caco-2 Cell Viability by MTT and LDH Assay

**Figure 3:**
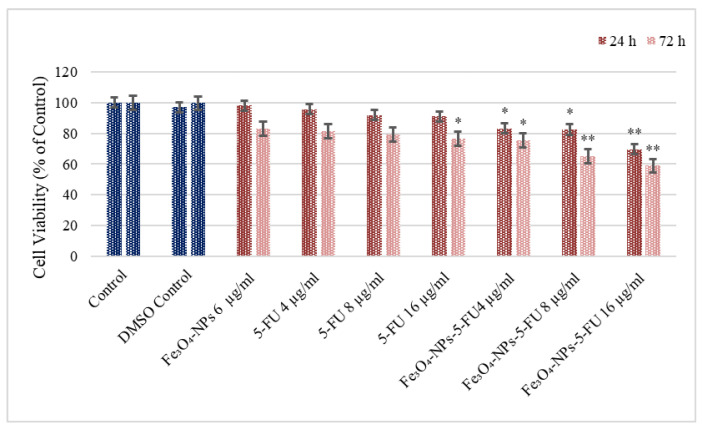
Cell viability was measured with an MTT assay (n = 6). The effect of Fe_3_O_4_0-NPs, 5-FU, and Fe_3_O_4_-NPs + 5-FU on Caco-2 cells’ viability. Cells were cultured in 96-well plates and treated with Fe_3_O_4_-NPs 6 μg/mL, 5-FU (4, 8, and 16 μg/mL), and the Fe_3_O_4_ NPs + 5-FU combination for 24 h and 72 h. The results are presented as the average of three separate experiments. Statistical significance: * *p* < 0.05; ** *p* < 0.01.

The effect of various treatments on Caco-2 cell viability was determined by the MTT assay after 24 and 72 h of treatment (Figure 3). Cell viability was considered as 100% in the control (negative control) and is expressed as a percentage of that of the control for all other treatments. Notably, DMSO and the nonloaded Fe_3_O_4_ NPs at 6 µg/mL did not affect the viability of Caco-2 cells. Treating the cells for 24 h with 5-FU 16 µg/mL exerted a nonsignificant 10% decrease in viability (*p* = NS), and the reduction (34%) was statistically significant after 72 h of treatment (*p* < 0.05). The effects of the Fe_3_O_4_-NPs + 5-FU combination on cell viability were more prominent. Thus, treating cells for 24 h with Fe_3_O_4_ NPs + 5-FU (16 µg/mL) decreased their viability to 31% (*p* < 0.05), whereas treating Caco-2 cells with Fe_3_O_4_ NPs + 5-FU (16 µg/mL) for 72 h resulted in a substantial decrease in their viability (41%) (*p* < 0.01).

**Figure 4:**
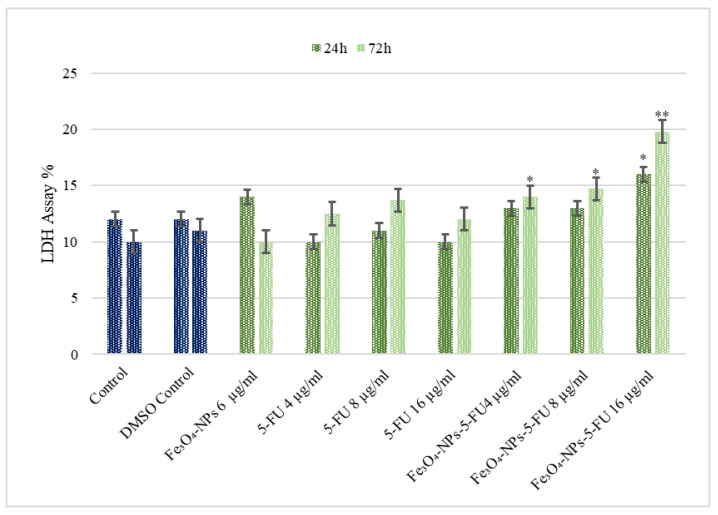
The effect of Fe_3_O_4_0-NPs, 5-FU, and Fe_3_O_4_-NPs + 5-FU on Caco-2 cell LDH activity (n = 6). Cells were cultured in 96-well plates and treated with Fe_3_O_4_-NPs 6 μg/mL, 5-FU (4, 8, and 16 μg/mL), and combinations of Fe_3_O_4_ + 5-FU NPs for 24 h and 72 h, and LDH activity was determined. The results represent the average of three separate experiments. Statistical significance is represented as * *p* < 0.05; ** *p* < 0.01.

Because LDH is released by necrotic cells, it is an excellent metabolic marker of cell viability. The effect of various treatments on Caco-2 cell LDH activity was determined by utilizing an LDH kit (Figure 3). The measured LDH activity of treated cells expressed as a percent of the standard (designated as 100%) is presented in Figure 4. Treating the cells with only Fe_3_O_4_-NPs and different concentrations of 5-FU did not affect their LDH activity. However, an increase in LDH activity, correlated with cell death, was demonstrated in cells treated with a combination of Fe_3_O_4_-NPs + 5-FU (8 µg/mL) for 72 h (*p* < 0.05) and cells treated with Fe_3_O_4_-NPs + 5FU (16 µg/mL) for 24 and 72 h, (*p* < 0.05 and *p* < 0.01), respectively. These data demonstrate that combining 5FU with Fe_3_O_4_-NPs significantly increased the active drug cytotoxic effect, even at concentrations below the active drug range.

### 3.3. The Effect of Fe_3_O_4_-NPs, 5-FU and Fe_3_O_4_-NPs + 5-FU on Caco-2 Cells Redox State 

**Figure 5:**
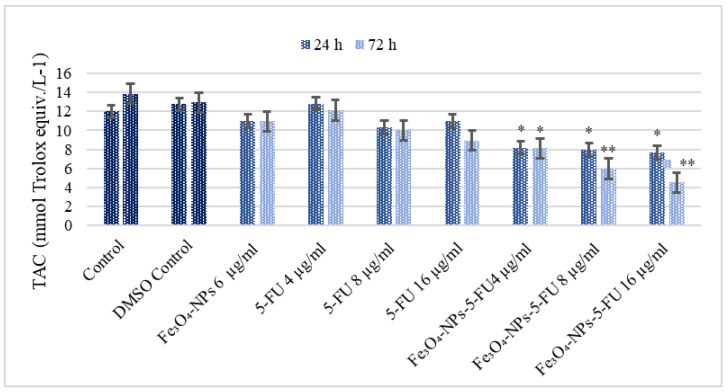
The effect of Fe_3_O_4_0-NPs, 5-FU, and Fe_3_O_4_-NPs + 5-FU on Caco-2 cells’ TAC (n = 6). Cells were cultured in 96-well plates and treated with Fe_3_O_4_ NPs 6 μg/mL, 5-FU (4, 8, and 16 μg/mL), and Fe_3_O_4_ + 5-FU NPs combinations for 24 h and 72 h, and TAC determined. The results represent the average of three separate experiments. Statistical significance: * *p* < 0.05; ** *p* < 0.01.

The Caco-2 cell TAC values, determined spectrophotometrically, were 12.01 and 13.84 mmol Trolox equiv/L, respectively (Figure 5). Treatment with Fe_3_O_4,_ NPs, and different concentrations of 5-FU did not affect these cells’ TAC. However, treatment with the loaded Fe_3_O_4_-NPs + 5-FU significantly decreased the Caco-2 cell antioxidant status in a time- and concentration-dependent manner (Figure 5).

**Figure 6:**
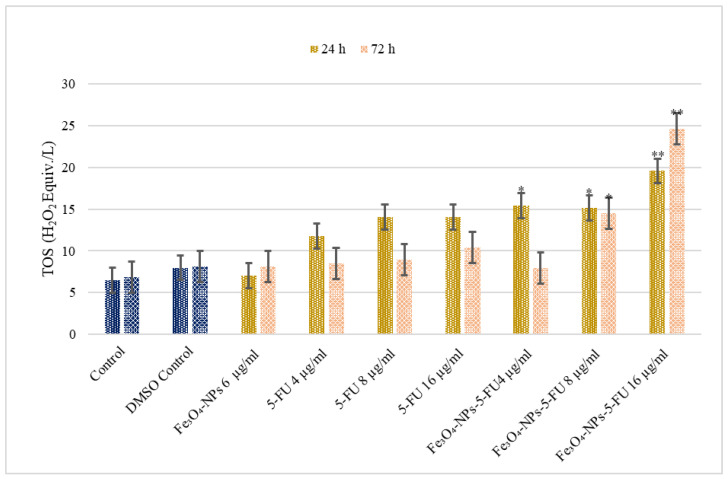
The effect of Fe_3_O_4_0-NPs, 5-FU, and Fe_3_O_4_-NPs + 5-FU on Caco-2 cells TOS levels (n = 6). Cells were cultured in 96-well plates and treated with Fe_3_O_4__NPs 6 μg/mL, 5-FU (4, 8, and 16 μg/mL), and combinations of Fe_3_O_4_ + 5-FU NPs for 24 h and 72 h, and TOS determined. The results represent the average of three separate experiments. Statistical significance: * *p* < 0.05; ** *p* < 0.01.

In correlation with the TAC results, the combined Fe_3_O_4_-NPs + 5-FU treatments (Figure 6) was found to increase Caco-2 cell TOS levels, dependent on time and concentration. The most pronounced effects were obtained after 72 h of treatment with Fe_3_O_4_-NPs + 5U 8 µg/mL/5-FU 16 µg/mL (*p* < 0.01).

### 3.4. The Effect of Fe_3_O_4_-NPs, 5-FU and Fe_3_O_4_-5-FU NPs on PTEN and IL-10 Gene Expression

**Figure 7:**
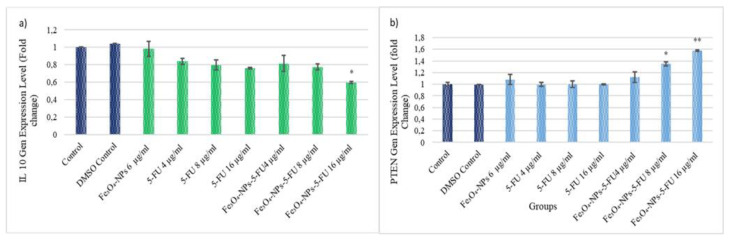
The effect of Fe_3_O_4_0-NPs, 5-FU, and Fe_3_O_4__NPs + 5-FU on PTEN and IL-10 gene expression. (**a**) IL-10 gene level; (**b**) PTEN gene level (n = 3). Cells were cultured in 96-well plates and treated with Fe_3_O_4__NPs 6 μg/mL, 5-FU (4, 8, and 16 μg/mL), and combinations of Fe_3_O_4_ + 5-FU NPs for 72 h. The results represent the average of three separate experiments. Statistical significance: * *p* < 0.05; ** *p* < 0.01.

PTEN and IL-10 gene expression levels were measured with real-time PCR analysis at the 72 h point of various treatments. This approach demonstrated that the Fe_3_O_4_-NPs + 5-FU (8 µg/mL) and Fe_3_O_4_-5-NPs + FU (16 µg/mL) treatments significantly upregulated PTEN expression (*p* < 0.05 and *p* < 0.01, respectively). A 1.34-fold increase for the Fe_3_O_4_-NPs + 5-FU (8 µg/mL) and a 1.57-fold for the Fe_3_O_4_-NPs + 5-FU (16 µg/mL) treatments were determined (Figure 7a).

On the other hand, the expression of the immunosuppressive IL-10 gene level was significantly downregulated in Caco-2 cells exposed to Fe_3_O_4_-NPs + 5-FU (16 µg/mL), with a 0.59 decrease (*p* < 0.05) (Figure 7b).

## 4. Discussion

Over the past decades, remarkable advances have occurred in nanotechnology, particularly nanomedicine, focusing, among others, on novel cancer therapeutics [35]. NPs can accumulate in cells without being recognized by *p*-glycoproteins, one of the primary mediators of multidrug resistance, resulting in increased intracellular concentrations of drugs [36]. Notably, NP carriers exhibit intrinsic abilities affecting cancer and immune cell biological functions [37]. Therefore, our study examined the synergistic effect of Fe_3_O_4_-NPs and 5-FU on Caco-2 colon cancer cell viability, oxidative stress, and oncogene expression. 

Previous studies have shown an ambiguous effect of iron oxide NPs on cell biological functions, dependent on cell type and concentration utilized. Thus, it was shown that iron oxide NPs could induce the cellular inflammatory response and increase the secretion of proinflammatory cytokines in human or mouse cells [37,38]. Lately, they have been approved by the FDA, and their beneficial effects on cell physiology have been suggested [13]. However, Fe_3_O_4_/composites were also shown to facilitate various active drugs’ cytotoxic and immunomodulatory properties [39]. Thus, the peroxidase-like activity of Fe_3_O_4_ and carbon NPs was found to facilitate ascorbic-acid-induced oxidative stress and to incur specific damage to PC-3 prostate cancer cells [40]. Furthermore, increased ROS production generates oxidative stress within the cells and cell apoptosis, resulting in PC-3 tumor cell growth inhibition [40]. Moreover, composite NPs can induce mitochondrial membrane alteration, DNA damage, cytokine production associated with oxidative stress, and apoptosis-correlated cell death [41]. 

A separate research direction is the regulation of magnetic fields, as various studies have shown that the discrete modulation of these fields can inhibit the proliferation of cancer cells and tumor growth [42,43]. Furthermore, due to the promoting effect of iron metabolism on ROS production, increased concentrations of iron-based NPs in cancer cells enhance their exposure to the local magnetic field and cellular death [44,45].

Our study showed that Fe_3_O_4_ NPs did not negatively affect Caco-2 cell viability, oxidative stress, or oncogene expression. However, together with 5-FU, Fe_3_O_4_ NPs acted synergistically, and the combination exerted cytotoxic, immunomodulatory, and oxidative-stress-promoting effects at a concentration at which the active drug does not affect these cell functions. Notably, LDH is a cytotoxic marker as its release is enhanced due to cell necrosis. In this study, combined Fe_3_O_4__NPs + 5-FU strongly increased LDH activity, correlated with the upregulation of cell death. Furthermore, TOS increase and TAC attenuation were evident after combined Fe_3_O_4__NPs + 5-FU treatment. Notably, the effects were exerted only after combined Fe_3_O_4__NPs + 5-FU treatment, as treating cells with only 5-FU at the same concentrations did not affect these parameters of cell homeostasis.

PTEN is a well-established tumor suppressor, and alterations in its expression/activity are correlated with tumorigenesis [46]. Moreover, as PTEN controls polarity in normal epithelial cells, loss of this protein plays a critical role in the invasion and metastasis of various cancer types, including colon cancer [47,48]. Several studies have established a negative correlation of PTEN expression with colon cancer progression due to its vital role in inhibiting the malignant transformation of intestinal epithelial cells [49]. A similar association was determined with the dysregulation of PTEN-binding partners [50,51,52]. In the present study, the combined Fe_3_O_4__NPs + 5-FU significantly increased this gene expression. Notably, PTEN acts as a negative regulator of the PI3K/Akt signaling pathway and was shown to affect many processes deregulated in tumorigenesis, such as cell survival, proliferation, migration, and invasion [53]. Indeed, inhibition of the PI3K/Akt pathway induces programmed cell death in different cell lines [54]. Recently, patients presenting PTEN hamartoma tumor syndrome were advised to employ earlier surveillance for colon cancer due to an increased risk of early onset [55]. In the present study, Fe_3_O_4_-NPs in combination with 5-FU increased PTEN gene expression. Moreover, in an in vivo prostate cancer model, a NP-mediated increase in PTEN led to disease remission, highlighting the importance of this gene in tumorigenesis and defining it as a promising therapeutic target and progression marker [56]. 

IL-10 is a versatile immunosuppressive cytokine with immunomodulatory functions [20]. Thus, IL-10 increases tumor cell survival, proliferation, and metastasis by controlling antitumor immunity. Indeed, IL-10 exerts suppressive effects on effector immune cells, including potent antitumor cytotoxic NK and CD8 T cells [21]. The immunosuppressive functions of IL-10 are exercised through the Jak1/STAT3 pathway. Moreover, IL-10 suppresses the level of proinflammatory cytokines, including IL-1β [57]. IL-10 exhibits a role in colon cancer progression as increased levels of IL-10 facilitated liver metastasis in a mouse model [55]. Reprogramming the colon cancer tumor environment by silencing IL-10 expression resulted in dendritic-cell-dependent activation of the antitumor response [56]. This is a significant achievement, especially as dendritic cells (DCs) have a key role in triggering antitumor immune responses [58]. Madhubala et al. [59], in their study on titanium dioxide NPs’ effects in a leukemia cell line, observed that the expression of IL-10 significantly decreased. In the present study, Fe_3_O_4_-NPs + 5-FU treatment significantly reduced the IL-10 release of colon cancer cells. 

Thus, this study shows that the combined administration of Fe_3_O_4_ and 5-FU NPs will reduce the dose of the drug required to achieve pronounced antitumor activity. The latter effect is a significant result since 5-FU has a pronounced toxicity, which can be reduced due to its immobilization, for example, using metal–organic frameworks [60,61,62]. Therefore, the role of NPs is not only to provide suitable dynamics for 5-FU release in the event of its immobilization but also to eliminate the barrier associated with penetration through the cell membrane. A similar synergistic effect was described when platinum NPs were administered together with nonimmobilized doxorubicin to U2OS osteosarcoma cells. In this model, cotreatment significantly increased the drug’s effectiveness compared with pure doxorubicin at a similar dose [63]. This was explained by an increase in oxidative stress in the presence of platinum NPs [63], which we also noted with the combined introduction of Fe_3_O_4_ and 5-FU nanoparticles in the present study. On the other hand, NP treatment promotes the activation of endocytosis [64], which can also promote the penetration of 5-FU through cell membranes. Therefore, administering anticancer drugs, even without their preliminary immobilization, together with NPs, can significantly increase their cytostatic activity. Furthermore, in vivo and in vitro experiments for the characterization of Fe_3_O_4_ nanoparticles/active drug effects on specific cells/tissues are in order.

## 5. Conclusions

Magnetite NPs penetrate (passive delivery) due to increased vascular permeability and weakened lymphatic drainage of cancer tissues. Moreover, magnetite NPs are easily uptaken and accumulate in cancer cells due to their small size. Our study showed that the combined Fe_3_O_4__NPs + 5-FU, through a synergistic effect, significantly reduced Caco-2 cell viability at a concentration at which the active drug did not induce an effect. Likewise, the combined treatment, but not the solitary components, facilitated oxidative stress correlated with the decreased viability of Caco-2 cells. Moreover, we determined that combined Fe_3_O_4__NPs + 5-FU treatment decreased IL-10 levels and enhanced the expression of the oncogene-suppressor PTEN. Our data show that Fe_3_O_4__NPs + 5-FU exhibit significant antitumor effects at low concentrations of the active drug. Further studies are needed to fully elucidate the molecular mechanisms involved. 

## Data Availability

Raw data is available on request.
